# Effectiveness and safety of early versus late caffeine therapy in managing apnoea of prematurity among preterm infants: a retrospective cohort study

**DOI:** 10.1007/s11096-022-01437-0

**Published:** 2022-08-01

**Authors:** Won Zi Yun, Yaman Walid Kassab, Liew Mei Yao, Norliyana Khairuddin, Long Chiau Ming, Muhammad Abdul Hadi

**Affiliations:** 1 Department of Pharmacy, Putrajaya Hospital, Ministry of Health Malaysia, 62250 Putrajaya, Malaysia; 2 College of Pharmacy, National University of Science and Technology, Muscat, Oman; 3 Department of Pharmacy, Serdang Hospital, Ministry of Health Malaysia, Putrajaya, Malaysia; 4 PAP Rashidah Sa’adatul Bolkiah Institute of Health Sciences, Universiti Brunei Darussalam, Gadong, Brunei Darussalam; 5 College of Pharmacy, Qatar University, QU Health, Doha, Qatar

**Keywords:** Caffeine, Metabolic bone disease, Neonatal Intensive Care Unit, Premature infant, Xanthine

## Abstract

**Background:**

Early administration of intravenous (IV) caffeine (initiation within 2 days of life) is an effective treatment strategy for the management of apnoea of prematurity among infants. However, the safety and effectiveness of early administration of oral caffeine treatment is not be fully established.

**Aim:**

We aimed to compare the effectiveness and safety of early versus late caffeine therapy on preterm infants’ clinical outcomes.

**Method:**

A retrospective matched cohort study was conducted using data of patients admitted to neonatal intensive care units of two tertiary care hospitals between January 2016 and December 2018. The clinical outcomes and mortality risk between early caffeine (initiation within 2 days of life) and late caffeine (initiation ≥ 3 days of life) were compared.

**Results:**

Ninety-five pairs matched based on gestational age were included in the study. Compared to late initiation, preterm infants with early caffeine therapy had: a shorter duration of non-invasive mechanical ventilation (median 5 days vs. 12 days; *p* < 0.001); shorter length of hospital stay (median 26 days vs. 44 days; *p*  < 0.001); shorter duration to achieve full enteral feeding (median 5 days vs. 11 days; *p* < 0.001); and lower frequency of bronchopulmonary dysplasia (BPD) (4.5% vs. 12.9%; *p* = 0.045). They also had a reduced risk of osteopenia of prematurity (OP) (OR 0.209; 95% CI 0.085–0.509; *p* = 0.001).

**Conclusion:**

Early oral caffeine therapy can potentially improve respiratory outcomes among infants with apnoea of prematurity. However, an increase in mortality associated with early caffeine therapy requires further investigation.

**Supplementary Information:**

The online version contains supplementary material available at 10.1007/s11096-022-01437-0.

## Impact statements

- Early administration of oral caffeine (initiation within 2 days of life) can be an effective treatment strategy for the management of apnoea of prematurity among infants, especially in settings where IV caffeine is not available.
- Safety of early administration of oral caffeine treatment cannot be fully established and requires further investigation using well-designed randomized controlled trials.
- Infant developmental milestones need to be carefully monitored among perterm infants who had received oral caffeine.

## Introduction

Preterm birth complications are the leading cause of mortality among under-5 years old, accounting for approximately 16% of 5.9 million under-5 deaths [1]. Apnoea of prematurity (AOP) is a preterm complication due to a developmental disorder that frequently affects preterm infants [2]. AOP is defined as an interruption of breathing for more than 20 s, or a shorter respiratory cessation period but associated with oxygen desaturation and/or bradycardia in preterm infants younger than 37 weeks of gestation [3]. Prolonged apnoea, bradycardia and reduced cerebral blood flow may lead to hypoxic-ischaemic harm to the infant’s developing brain [4].

Methylxanthines such as caffeine citrate or theophylline are the primary pharmacological agents used to treat AOP [5]. Caffeine citrate is the preferred therapy for AOP due to its pharmacokinetic profile of long half-life and good oral absorption [6, 7]. The Caffeine for Apnoea of Prematurity trial concluded that early caffeine therapy (initiation within 2 days of life) is associated with fewer intubation episodes and reduced use of positive pressure ventilation compared to late caffeine therapy (initiation from day 3 of life onwards) [8]. Caffeine has interethnic pharmacokinetic differences. CYP1A2 is the main caffeine metabolism enzyme encoded by the CYP1A2 gene which has gene polymorphism leading to interindividual variability [9, 10]. Although a recent study concluded that there was no significant association between CYP1A2 gene variation and caffeine pharmacokinetic parameters [10], the genetic polymorphisms in caffeine targeting the adenosine receptor induced different clinical responses to caffeine therapy [11, 12].

In Malaysia**,** the fourth report of the National Obstetrics Registry stated that 8.9% of the total number of deliveries in 15 state hospitals were preterm in 2017 [13]. The routine practice for managing AOP in a neonatal intensive care unit (NICU) in Malaysia is to initiate methylxanthines prophylatically with a loading dose of IV aminophylline, followed by a maintenance dose of IV aminophylline, and then to switch to oral caffeine after initiation of oral feeding in infants with gestational age less than 32 weeks. For those infants with gestational age between 32 and 34 weeks, treatment is given if present with apnoea symptoms. This practice has been adopted due to the non-availability of caffeine citrate injections in the majority of the Malaysian government hospitals, as the drug is not listed in the Malaysian Ministry of Health (MOH) National Drug Formulary, and there is a lack of safety data on the early initiation of an oral caffeine solution. Hospital pharmacists make an oral caffeine solution from caffeine powder as an alternative to caffeine citrate injections. Therefore, the caffeine therapy initiation is usually onwards from day three of life, which is classified as late caffeine therapy. Owing to the fact that caffeine is the preferred therapy in the management of AOP [3], some NICUs adopted the practice of off-label early oral caffeine therapy in preterm infants. The safety of administering oral caffeine to preterm infants before initiation of oral feeding is uncertain, however, as the published studies on early caffeine therapy were carried out with intravenous administration of caffeine.

### Aim

In order to inform local practice and policy with regards to the management of AOP, this multicentre study was carried out by a group of clinical pharmacists to investigate the effectiveness and safety of early oral caffeine therapy on preterm infants’ clinical outcomes.

### Ethics approval

This research project was given ethical approvalby the Ministry of Health Medical Research Ethical Committee (MREC) [Approval number: NMRR-19-423-45654; reference number KKM/NIHSEC/P19-769(5), dated 26 April 2019]. The study adhered fully to the guidelines outlined in the Declaration of Helsinki.

## Method

This retrospective cohort study was carried out at two Level III NICUs in Malaysia. Level III (subspecialty) NICUs have the personnel (neonatologists, neonatal nurses, respiratory therapists) and equipment to provide life support for as long as needed. The study population included all preterm infants born at less than 37 weeks gestational age and admitted to an NICU within 24 h of life between January 2016 and December 2018. Infants with major congenital malformation, treated with an inappropriate caffeine dosing regimen (not adhering to local practice protocol of oral caffeine dose range of 5–10 mg/kg daily), and infants with missing data on caffeine therapy were excluded from the study.

### Sample size

Sample size was calculated using two population proportions formulae [14, 15]. The sample size was calculated based on the incidence of diagnosis of bronchopulmonary dysplasia (BPD). Previous data indicated that the proportion of BPD in the early group was 0.20 and the proportion of the late group was 0.346 [14]. Thus, the minimum sample size of 145 samples per group was required with 80% power and a *p*-value of 0.05. According to finite population of 417 patients in Hospital Putrajaya and 363 patients in Hospital Serdang respectively, the corrected sample size of patients for Hospital Putrajaya and Hospital Serdang was 108 and 104 samples respectively. With an additional of 20% dropout samples due to incomplete data, the sample size was at least 130 samples per group [15].

#### Data collection

A random sampling method was conducted to select samples for this study. The relevant patients’ registry numbers extracted from the hospital databases were randomly selected by using random numbers generated by the Microsoft Excel program. All data were extracted from the electronic medical records database. Data were collected until death or discharge from the NICU. The flow of patient selection during the study is shown in Figure S1. Based on the timing of caffeine therapy initiation, preterm infants were divided into 2 groups: early caffeine therapy (caffeine initiation within 2 days of life) and late caffeine therapy (caffeine initiation ≥ 3 days of life). The baseline demographics and clinical outcomes were compared between the two groups. A structured pilot-tested data collection form was used to standardize data collection. The outcomes measured included: BPD, ventilation days, length of hospital stay (LOS), average weight gain, osteopenia of prematurity (OP), death, duration to reach full enteral feeding, and necrotising enterocolitis.

### Outcome measures: key definitions

BPD was defined as the need for a continuous supplemental oxygen concentration > 21% by nasal cannula, nasal catheter, face mask or respiratory support for at least 28 continuous days or by corrected age of 36 weeks, or more than 56 days of postnatal age for infants born after more than 32 weeks gestation [16].

The duration of mechanical ventilation was defined as the total number of days preterm infants required mechanical ventilation support, whereas non-invasive respiratory ventilation support includes continuous positive airway pressure (CPAP), bilevel positive airway pressure (BiPAP), or high-flow nasal cannula (HFNC) [17].

Extubation failure was defined as the need to resume mechanical ventilation within less than 72 h. Reintubation was defined as the need for reintubation at any time after 72 h of extubation [18].

The length of hospital stay was defined as the total number of NICU admission days.

Necrotising enterocolitis was defined according to Bell criteria: it was diagnosed clinically or radiologically by a ward neonatologist [19].

OP was diagnosed biochemically with the elevation of serum ALP > 500U/L or serum phosphate < 1.8 mmol/L [20, 21].

### Data analysis

Propensity score matching (PSM) was used minimize the impact of baseline characteristic differences between early and late caffeine therapy groups on the treatment outcomes[22]. The two groups were matched based on gestational age. All data were analyzed using the IBM Statistical Package for Social Sciences (SPSS) version 24.0 software. Comparison of numerical data (such as LOS, duration of mechanical ventilation etc.) between early caffeine therapy and late caffeine therapy groups that were normally distributed were analyzed using the independent *t*-test, while the Mann–Whitney test was used if the data for two independent groups were not normally distributed. To study the association between two sets of categorical data (such as BPD and study group, OP and study group etc.), Pearson's Chi-square test for Independence was used, while Fisher's exact test was used if the assumptions of Pearson's Chi-square test for independence were violated. Binary logistic regression was used to test for significant predictors among the independent variables. The risk was expressed as an odds ratio (OR) with 95% CI. A 2-sided *p*-value < 0.05 was considered statistically significant for all tests.

## Results

### Baseline characteristics

A total of 190 preterm infants meeting the inclusion criteria for the study were PS matched in 1:1 ratio based on gestational age. The mean gestational age between early and late caffeine therapy group was similar (31.23 weeks vs. 30.61 weeks; *p*-value = 0.055).. The median postnatal age caffeine therapy initiation was 1 day (range 0–1 day of life) and 13 days (range 6–13 days of life) in early and late caffeine therapy groups, respectively (Table 1). Study results showed that preterm infants treated with early caffeine had higher mean birth weight compared to those in the late caffeine group (1.53 kg vs. 1.33 kg; *p*-value = 0.001). There were no significant differences in ethnicity, gender, Apgar score, mode of delivery, maternal chorioamnionitis, maternal gestational diabetes and hypertension between the two groups. Antenatal steroid administration was higher in the early caffeine group. Nearly all mothers in the early caffeine commencement group were administered an antenatal steroid following the standard protocol of total dose of intramuscular administration of Dexamethasone 24 mg prior to delivery compared to only three-fifths of mothers in the late caffeine group.

**Table 1:** Demographics and baseline clinical characteristics of preterm infants included in the study

Characteristics	Caffeine group		*p* value
	Early caffeine (n = 95)	Late caffeine (n = 95)	
Gestational age (weeks)	31.23 ± 2.30	30.61 ± 2.14	0.055
Birth weight (gram)	1.53 ± 0.40	1.33 ± 0.38	0.001
Gender			
Male	52 (54.7%)	45 (47.4%)	0.310
Female	43 (45.3%)	50 (52.6%)	
Ethnicity			
Malay	74 (77.9%)	75 (78.9%)	0.057
Chinese	13 (13.7%)	4 (4.2%)	
Indian	5 (7.4%)	9 (9.5%)	
Others	3 (5.3%)	7 (7.4%)	
Apgar score			
1 min	7.200 ± 2.31	7.096 ± 2.47	0.765
5 min	10 (8–10)	10 (9–10)	0.493
Mode of delivery			
Spontaneous vaginal delivery	26 (27.4%)	36 (37.9%)	0.122
Caesarean section	69 (72.6%)	59 (62.1%)	
Small for gestational age	10 (10.5%)	10 (10.5%)	1.000
Maternal chorioamnionitis	3 (3.2%)	7 (7.4%)	0.194
Maternal GDM/T2DM	23 (24.2%)	17 (17.9%)	0.286
Maternal HPT/PE/Eclampsia*	37 (38.9%)	21 (22.1%)	0.012
Maternal GDM + HPT	11 (11.6%)	4 (4.2%)	0.060
Antenatal steroid therapy*	94 (98.9%)	61 (64.2%)	< 0.001
Maternal parity*			
Low multiparity	83 (87.4%)	66 (69.5%)	0.003
Grand multipara	12 (12.6%)	29 (30.5%)	
Intubation on day 1 of life	61 (64.2%)	67 (70.5%)	0.353
Surfactant therapy*	42 (44.2%)	57 (60.0%)	0.029
Postnatal age when Caffeine initiated (days)*	1 (0)	13 (10–16)	< 0.001
Duration of caffeine therapy (days)	13 (8–24)	16 (7–28)	0.710
Caffeine Cumulative Dose (mg/kg)*	85 55–172.5)	160 (70–280)	0.008
TPN therapy*	30 (31.6%)	62 (65.3%)	< 0.001
TPN duration (days)	10 (13.3)	11 (9–15)	0.066

Data expressed as mean ± SD, median (interquartile range) or frequency (percentage)

(GDM: Gestational Diabetes Mellitus, T2DM: Type II Diabetes Mellitus, HPT: hypertension, PE: Pre-eclampsia, TPN: Total Parenteral Nutrition)

*p < 0.05 is statistically significant

### Respiratory outcomes

Respiratory outcomes were studied among 182 infants, excluding those preterm infants who passed away prior to hospital discharge.. Preterm infants with early caffeine therapy had a shorter duration of non-invasive mechanical ventilation and were able to be weaned off oxygen support 9 days earlier than those in the late caffeine therapy group (*p*-value < 0.001) (Table 2). The number of infants who developed BPD was significantly lower in the early caffeine group compared with the late caffeine group (4.5% vs. 12.9%; *p*-value = 0.045). We analyzed 131 intubated preterm infants. The results showed that early caffeine therapy non-significantly reduces extubation failure among preterm infants as compared to late caffeine therapy (*p*-value = 0.690), with a difference of 2%. Preterm infants in both early and late caffeine therapy groups had similar duration of invasive mechanical ventilation (median 2, IQR (3), *p*-value = 0.219).

**Table 2:** Comparison of respiratory outcomes in preterm infants administered with early caffeine and late caffeine

Respiratory outcomes	Caffeine group		*p*-value
	Early caffeine (n = 89)	Late caffeine (n = 93)	
Postnatal age at first extubation	2 (1–3)	2 (2–4)	0.172
Extubation failure	8 (9%)	10 (10.8%)	0.690
Documented days of apnoea	18 (18.9%)	10 (10.5%)	0.624
Duration of invasive mechanical ventilation (days)	2 (0–3)	2 (0–3)	0.219
Duration of non-invasive mechanical ventilation (days)*	5 (3–11.7)	12 (6.5–43)	< 0.001
Postnatal age to room air (days of life)*	6 (4–15.7)	15 (7–47.5)	< 0.001
Discharged home on oxygen	1 (1.1%)	0 (0%)	0.489
Postnatal steroids used	4 (4.5%)	10 (10.8%)	0.113
BPD*	4 (4.5%)	12 (12.9%)	0.045

Data expressed as mean ± SD, median (interquartile range) or frequency (percentage)

*BPD* bronchopulmonary dysplasia

*p < 0.05 is statistically significant

Direct multivariate logistic regression analysis was performed to identify the factors that predict BPD among preterm infants (Table 3). The model contained five independent variables (gestational age, birth weight, small for gestational age, administered antenatal steroid and caffeine administration timing). Early caffeine therapy reduced the risk of developing BPD by nearly 1.5 times compared to late caffeine therapy among preterm infants, though this result was not statistically significant (OR 1.41; 95% CI 0.296–6.757, *p*-value = 0.664).

**Table 3:** Predictors of BPD in preterm infants by logistic regression analysis (n = 182)

Variables	Univariate Crude OR (95% CI)	*p* value	Multivariate Adjusted OR (95% CI)	*p* value
Gestational age	0.512 (0.388–0.675)	< 0.001	0.581 (0.321–1.050)	0.072
Birth weight (kg)	0.002 (0.000–0.028)	< 0.001	0.019 (0.000–2.108)	0.099
Small for gestational age^a^	2.163 (0.557–8.405)	0.265	7.103 (0.422–119.502)	0.173
Antenatal steroid^b^	0.662 (0.200–2.194)	0.500	0.228 (0.038–1.356)	0.104
Early caffeine therapy^c^	0.318 (0.098–1.025)	0.055	0.707 (0.148–3.373)	0.664

*OR* odds ratio

^a^Compared to non-SGA

^b^Compared to no antenatal steroid

^c^Compared to late caffeine therapy

*p < 0.05 is statistically significant

### Early caffeine and neonatal outcomes

Preterm infants treated with early caffeine achieved full enteral feeding 6 days earlier and were discharged home 18 days earlier than those in the late caffeine therapy group (*p-*value < 0.001). On the other hand, early caffeine therapy was associated with significantly lower daily weight increment (*p-*value < 0.001). There was a higher proportion of preterm infants’ death in the early caffeine group compared to the late caffeine therapy group although this result was not statistically significant (6.3% vs. 2.1%, *p-*value = 0.279) (Table 4).

**Table 4:** Comparison of neonatal outcomes in preterm infants administered with early caffeine and late caffeine

Neonatal outcomes	Caffeine group		*p* value
	Early caffeine (n = 89)	Late caffeine (n = 93)	
Death before hospital discharge (n = 190)	6 (6.3%)	2 (2.1%)	0.279
Length of hospital stay (days)*	26 (17–41.5)	44 (35–78.5)	< 0.001
Duration required to reach full enteral feeding (days)*	5 (3–7)	11 (9–14)	< 0.001
Average weight gain (g/day)*	8.07 ± 6.71	12.02 ± 5.57	< 0.001
Osteopenia of prematurity*	7 (7.9%)	27 (29%)	< 0.001
Episodes of caffeine withhold for suspected caffeine induced tachycardia	1 (1.1%)	1 (1.1%)	1.000
Necrotising enterocolitis	2 (2.2%)	2 (2.2%)	1.000

Data expressed as mean ± SD, median (interquartile range) or number (percentage)

*p < 0.05 is statistically significant

### Adverse effects

Preterm infants treated with late caffeine therapy were more likely to be diagnosed with OP when compared to those treated with early caffeine initiation (29% vs. 7.9%, *p-*value < 0.001) (Table 4). The proportions of necrotising enterocolitis and tachycardia in these two groups were identical (*p-*value = 1).

A direct multivariate logistic regression was performed to assess the factors that predict OP among preterm infants. The final model contained 5 independent variables (gestational age, birth weight, TPN duration, cumulative caffeine dose and caffeine administration timing). The odds of a preterm infant being diagnosed as OP were about 2 times higher for the late caffeine therapy group compared to the early caffeine therapy group, though the association was not statistically significant (OR 0.532; 95% CI 0.156–1.820, *p*-value = 0.315) (Table 5).

**Table 5:** Predictors of osteopenia of prematurity in preterm infants by logistic regression analysis (n = 182)

Variables	Univariate Crude OR (95% CI)	*p-*value	Multivariate Adjusted OR (95% CI)	*p-*value
Gestational age (weeks)	0.697 (0.584–0.830)	< 0.001	1.315 (0.900–1.920)	0.156
Birth weight (kg)	0.019 (0.004–0.085)	< 0.001	0.264 (0.009–7.731)	0.439
TPN total duration (days)	1.179 (1.107–1.255)	< 0.001	1.106 (0.994–1.231)	0.064
Cumulative caffeine dose (mg/kg)	1.005 (1.003–1.007)	< 0.001	1.005 (1.001–1.009)	0.016
Early caffeine therapy^a^	0.209 (0.085–0.509)	0.001	0.532 (0.156–1.820)	0.315

^a^Compared to late caffeine therapy

*TPN* total parenteral nutrition

*p < 0.05 is statistically significant

More importantly, the odds of death prior to hospital discharge among preterm infants were about 8 times higher in the early caffeine therapy group compared to the late caffeine therapy group (OR 8.058; 95% CI 1.063–61.065, *p*-value = 0.043) (Table 6).

**Table 6:** Predictors of death before hospital discharge in preterm infants by logistic regression analysis (n = 190)

Variables	Univariate Crude OR (95% CI)	*p* value	Multivariate Adjusted OR (95% CI)	*p* value
Gestational age (weeks)	0.662 (0.494–0.887)	0.006	0.916 (0.615–1.365)	0.667
Birth weight (kg)	0.033 (0.003–0.407)	0.008	0.027 (0.001–1.110)	0.057
Antenatal steroid^a^	1.608 (0.191–13.507)	0.662	0.369 (0.027–5.064)	0.456
Early caffeine therapy^b^	3.135 (0.616–15.944)	0.169	8.058 (1.063–61.065)	0.043

*OR* Odds ratio

^a^Compared to no antenatal steroid

^b^Compared to late caffeine therapy

*p < 0.05 is statistically significant

## Discussion

The aim of this study was to compare the effectiveness and safety of early versus late caffeine therapy for the management of AoP on preterm infants’ clinical outcomes. This study adds to existing evidence on the safety and efficacy of oral caffience therapy and the time of initiation of oral caffeine. Our study findings are particularly useful in settings with limited availability of caffeine injections as this study provides safety evidence on the early commencement of oral caffeine in preterm infants.

Bronchopulmonary dysplasia is a chronic respiratory disease among preterm infants resulting in significant morbidity and mortality [23]. In this study, we found that early caffeine therapy was associated with a significantly lower incidence of BPD. The risk reduction associated with the early caffeine therapy was in line with the Caffeine for Apnoea of Prematurity trial that reported a 37% reduction in BPD compared to a 13% reduction in the late caffeine therapy group [8]. The proposed mechanism of caffeine in reducing the risk of BPD is caffeine blocking the Adenosine 2 receptor that leads to inflammation and remodelling in the lung [14]. Early caffeine therapy initiation may enhance lung function, reduce inflammation and thus prevent the development of BPD in preterm infants [24].

In this study, we noticed antenatal steroid administration was significantly higher in preterm infants treated with early caffeine therapy compared to late caffeine therapy. The wide variability in antenatal steroids between the two study centres is in concordance with the National statistics. The Malaysian National Neonatal Registry reported that there were marked differences in the use of antenatal steroids across the hospitals, ranging from 36.4 to 100.0% [19]. However, the significant difference in antenatal steroid administration practices between the two centres might not have significantly affected our study outcomes, particularly on BPD, as the previous research [25] showed no significant reduction in BPD with antenatal steroid therapy (RR 0.86, 95% CI 0.42–1.79).

In contrast to the Caffeine for Apnoea of Prematurity trial, Taha et al. concluded that early caffeine therapy increased the risk of necrotising enterocolitis in preterm infants (OR 1.41, 95% CI 1.04–1.91, *p*-value = 0.027) [24]. However, our data indicated that the incidence of necrotising enterocolitis was similar in both treatment groups. Earlier studies [26, 27] showed that caffeine therapy was a risk factor for feeding intolerance. In contrast, our data demonstrated early caffeine therapy was associated with a shorter duration to achieve full enteral feeding. Preterm infants need to develop skills in sucking–swallowing–breathing coordination to facilitate full enteral feeding [28]. We noticed that early caffeine improved respiratory outcomes and reduced the duration of mechanical ventilation and subsequent, reduction in breathing instability improved sucking–swallowing–breathing coordination in preterm infants, resulting in the early caffeine group achieving full enteral feeding earlier than those in the late caffeine group.

The risk of osteopenia of prematurity was inversely proportional to gestational age and directly related to TPN therapy. A study reported fractures in 30% of premature neonates with osteopenia of prematurity [29]. Our study reported that early caffeine therapy was associated with lower osteopenia of prematurity risk. Furthermore, the osteopenia of prematurity risk was strongly associated with an increment in cumulative caffeine dose in line with earlier results [29].. In this study, we collected data retrospectively, without taking into account maternal caffeine intake during the breastfeeding period or during pregnancy. Furthermore, in this study, the early caffeine therapy group, caffeine was prescribed at 5 mg/kg, compared to 10 mg/kg in the late caffeine therapy group. Nonetheless, both practices were in line with dose recommendations, as the optimum lowest effective dose of caffeine has yet to be investigated.

A randomized control trial on the benefit of early caffeine in weaning of mechanical ventilation among premature infants born at a gestational age between 23 and 30 weeks found a non-significant increase in mortality with early caffeine initiation [30]. The finding concurred with our study results. However, the mean gestational age of 26 weeks and the mean birth weight of less than 750 g in Amaro et al.'s [30] study were lower than our studied population. We further investigated all mortality cases in our study population. The deaths were due to sepsis and other complications of prematurity. None of the causes of death was directly related to caffeine therapy. Another possible contributing reason to the higher mortality in the early caffeine group is survival bias. In this study, the median postnatal age of preterm infants initiated with caffeine therapy in the late caffeine group was on day 13 of life, compared to day 1 of life in the early caffeine group. Preterm infants often have a lower survival rate during the first 24 h of life. The World Health Organization (WHO) reported that 75% of neonatal deaths occur during the first 7 days of life. Thus, it is possible that preterm infants in the early caffeine group have a lower survival rate compared to the late caffeine group who start caffeine therapy after passing through the high mortality risk period during the first week of life.

### Limitations

There were some limitations to the study owing to the retrospective study design and availability of data. Some relevant clinical data in patient monitoring (e.g. blood gas results) were not available in the hospital databases. This study did not include infants’ comorbidities. Patients’ clinical condition and concurrent drug therapy could be strong confounding factors affecting clinical outcomes, therefore the results need to be carefully interpreted.. Despite our attempts to match the two groups by gestational age, other baseline clinical characteristics were not matched statistically. The impact of baseline differences on study outcomes should be further investraged and a well-designed randomized controlled trial should be conducted to confirm the safety and effectiveness of early caffeine therapy among preterm infants with AOP. Furthermore, our study was never set out to identify optimal dose of oral caffeiene and future studies are required to investigate optimum caffeine duration and the lowest effective caffeine dose in the management of AOP. The long-term safety of caffeine therapy in preterm infants particularly on the growth and development should also be investigated.

## Conclusion

Overall, our study found that early caffeine therapy improved respiratory outcomes, shortened the duration of hospital stay and reduced the time taken to achieve full enteral feeding without increasing gastrointestinal adverse events in preterm infants. Moreover, early caffeine therapy was associated with lower weight gain and increased mortality risk. However, given the study design limitations, no definitive conculsions can be drawn on the safety and efficacy of oral caffeine. We suggest providing individualized treatment by weighing the benefits against the risks in terms of the appropriate timing of caffeine therapy in preterm infants until further study confirms the safety profile of early commencement of oral caffeine therapy.

## Supplementary Information

Below is the link to the electronic supplementary material.

Supplementary file1 (DOCX 27 kb)

## References

[1] World Health Organization. Preterm birth. 2018. Available from: https://www.who.int/news-room/fact-sheets/detail/preterm-birth. Accessed 07 Aug 2019.

[2] Henderson‐Smart DJ, Steer PA. Caffeine versus theophylline for apnea in preterm infants. Cochrane Database Syst Rev. 2000;(2):CD000273. doi: 10.1002/14651858.CD000273 10.1002/14651858.CD000273 10796190

[3] Eichenwald EC, Watterberg KL, Aucott S, et al. Apnea of prematurity. Pediatrics. 2016;137(1). 10.1542/peds.2015-3757 26628729

[4] Zhao J, Gonzalez F, Mu D (2011). Apnea of prematurity: from cause to treatment. Eur J Pediatr.

[5] Martin R, Garcia-Prats JA, Mallory GB. Management of apnea of prematurity – UpToDate. 2018 Available from: https://www.uptodate.com/contents/management-of-apnea-of-prematurity/print. Accessed 14 Nov 2018.

[6] Abdel-Hady H, Nasef N, Abd Elazeez Shabaan IN. Caffeine therapy in preterm infants. World J Clin Pediatr 2015;4(4):81. 10.5409/wjcp.v4.i4.81 PMC4637812 26566480

[7] Shrestha B, Jawa G (2017). Caffeine citrate: Is it a silver bullet in neonatology?. Pediatr Neonatol.

[8] Davis PG, Schmidt B, Roberts RS, et al. Caffeine for Apnea of Prematurity trial: benefits may vary in subgroups. J Paediatr 2010;156(3):382–7. 10.1016/j.jpeds.2009.09.069 19926098

[9] Lim MS, Son MJ, Shin JE (2017). Clinical pharmacokinetics of caffeine in Korean preterm infants with apnea of prematurity. Neonatal Med.

[10] Gao XB, Zheng Y, Yang F (2021). Developmental population pharmacokinetics of caffeine in Chinese premature infants with apnoea of prematurity: a post-marketing study to support paediatric labelling in China. Br J Clin Pharmacol.

[11] Kumral A, Tuzun F, Yesilirmak DC (2012). Genetic basis of apnoea of prematurity and caffeine treatment response: role of adenosine receptor polymorphisms: genetic basis of apnoea of prematurity. Acta Paediatr.

[12] He X, Qiu J-C, Lu K-Y (2021). Therapy for apnoea of prematurity: a retrospective study on effects of standard dose and genetic variability on clinical response to caffeine citrate in Chinese preterm infants. Adv Ther.

[13] Ministry of Health. Malaysia: National obstetrics registry; 2016–2017. p. 9.

[14] Park HW, Lim G, Chung S-H (2015). Early caffeine use in very low birth weight infants and neonatal outcomes: a systematic review and meta-analysis. J Korean Med Sci.

[15] Rubin A. Statistics for evidence-based practice and evaluation. Cengage Learning; 2012. p.107.

[16] Kair LR, Leonard DT, Anderson JM (2012). Bronchopulmonary dysplasia. Pediatr Rev.

[17] Lodha A, Seshia M, McMillan DD (2015). Association of early caffeine administration and neonatal outcomes in very preterm neonates. JAMA Pediatr.

[18] Costa ACDO, Schettino RDC, Ferreira SC (2014). Predictors of extubation failure and reintubation in newborn infants subjected to mechanical ventilation. Rev Bras Ter Intensiva.

[19] Ministry of Health. Malaysia: Malaysian National Neonatal Registry (NMRR); 2017.

[20] Backström M, Kouri T, Kuusela AL (2000). Bone isoenzyme of serum alkaline phosphatase and serum inorganic phosphate in metabolic bone disease of prematurity. Acta Pediatr.

[21] Abdallah EA, Said RN, Mosallam DS, et al. Serial serum alkaline phosphatase as an early biomarker for osteopenia of prematurity. Medicine. 2016;95(37). 10.1097/MD.0000000000004837 PMC5402581 27631238

[22] Walsh MC, Trentham-Dietz A, Newcomb PA (2012). Using propensity scores to reduce case-control selection bias. Epidemiology.

[23] Eichenwald EC, Stark AR. Bronchopulmonary dysplasia: Definition, pathogenesis, and clinical features - UpToDate. 2019. https://www.uptodate.com/contents/bronchopulmonary-dysplasia-definition-pathogenesis-and-clinical-features#H1. Accessed 14 Nov 2019.

[24] Taha D, Kirkby S, Nawab U (2014). Early caffeine therapy for prevention of bronchopulmonary dysplasia in preterm infants. J Matern Fetal Neonatal Med.

[25] Roberts D, Brown J, Medley N, et al. Antenatal corticosteroids for accelerating fetal lung maturation for women at risk of preterm birth. Cochrane Database Syst Rev.2017; 3(3):CD004454. doi: 10.1002/14651858.CD004454.pub3. 10.1002/14651858.CD004454.pub3 PMC6464568 28321847

[26] Gounaris AK, Grivea IN, Baltogianni M (2020). Caffeine and gastric emptying time in very preterm neonates. J Clin Med.

[27] Huang X, Chen Q, Peng W (2018). Clinical characteristics and risk factors for feeding intolerance in preterm infants. Zhong Nan Da Xue Xue Bao Yi Xue Ban.

[28] Dodrill P. Feeding difficulties in preterm infants. ICAN: Infant, Child, & Adolescent Nutrition. 2011;3(6):324–31.

[29] Ali E, Rockman-Greenberg C, Moffatt M (2018). Caffeine is a risk factor for osteopenia of prematurity in preterm infants: a cohort study. BMC Pediatr.

[30] Amaro CM, Bello JA, Jain D (2018). Early caffeine and weaning from mechanical ventilation in preterm infants: a randomized, placebo-controlled trial. J Pediatr.

