# Supplementary material for: Effectiveness and safety of early versus late caffeine therapy in managing apnoea of prematurity among preterm infants: a retrospective cohort study
Source: Int J Clin Pharm. 2022 Aug 1;44(5):1140–8. doi: 10.1007/s11096-022-01437-0 (PMC9618482; doi:10.1007/s11096-022-01437-0)
Supplement: Supplementary file 1 — Supplementary file1 (DOCX 27 kb) [file 11096_2022_1437_MOESM1_ESM.docx]

Total preterm infant admitted to NICU and prescribed with caffeine during January 2016 to December 2018

Hospital Serdang: 363 patients

Hospital Putrajaya: 417 patients

Total: 780 patients

Total preterm infants eligible for the study

Hospital Serdang: 130 patients

Hospital Putrajaya: 130 patients

Excluded patients

Hospital Serdang: 7 patients

- Transferred out: 2
- Incomplete data: 2
- Outborn: 3

Hospital Putrajaya: 5 patients

- Incomplete data: 2
- Outborn: 3

Total preterm infants included for the study

Hospital Serdang: 123 patients

Hospital Putrajaya: 125 patients

Early Caffeine Group (Case)

95 patients

Late Caffeine Group (Control)

95 patients

Sample collected according to sample size calculation

Matched according to gestational age
